# Attitudes and opinions of farmers in the context of environmental protection in rural areas in Poland

**DOI:** 10.1007/s10661-020-8133-5

**Published:** 2020-02-17

**Authors:** Arkadiusz Piwowar

**Affiliations:** Department of Economics and Organization of Food Economy, Faculty of Economy and Finance, Wroclaw University of Economics and Business, ul. Komandorska 118/120, 53-345 Wrocław, Poland

**Keywords:** Sustainable development, Environmental protection, Economics, Finance, Agriculture, Questionnaire surveys

## Abstract

This paper presents results of questionnaire surveys regarding the state of the natural environment in rural areas. The research was conducted on a sample of 1101 agricultural holdings in Poland. According to the analyses, agricultural producers are aware of environmental threats posed by irrational agricultural economy. Most respondents indicated the responsibility of farmers and other residents of rural areas as a basic condition for broadly understood environmental safety in rural areas in Poland. In the opinion of respondents, systems and programs for funding the replacement of heating boilers, thermomodernization of buildings, etc., are also important. There were significant differences in farmers’ declarations, taking into account the age and level of education of the respondents, as well as features of agricultural holdings (area and economic size of the agricultural holding).

## Introduction

Agriculture and food economy are indispensable sectors of the economy in every country. Food security and safe food are not just slogans but also a strategic need of societies (Ramankutty et al. 2018; Hidrobo et al. 2018). The primary purpose of agricultural activity is the production of raw materials and ready-made food products, which is associated with changes in ecosystems and may result in losses (damage) in the natural environment (Carvalho 2017; Kim et al. 2017). The threats may concern various elements of the natural environment: soil, water, and air (Stephens et al. 2018; Zhang et al. 2018; Lai 2017; Evans et al. 2019; Dzierzbicka-Głowacka et al. 2019). A high level of consumption of mineral fertilizers, especially nitrogen and phosphorus fertilizers, can cause adverse changes in soils and waters. Also, livestock production, especially intensive raising, may cause excessive external costs (emissions of gases, odors, problems with disposal of animal feces, etc.) (Piwowar 2018; Li et al. 2016; Wang et al. 2017; Mielcarek and Rzeźnik 2015).

The protection of the natural environment becomes an important element of the rational activities in the field of agricultural production more and more frequently (Pretty et al. 2018; Zilberman et al. 2018). Significant transformations in this matter took place in legal regulations and development programs in the area of agriculture, including the Common Agricultural Policy (Bureau and Swinnen 2018). The importance of sustainable development and low-carbon economy in the context of rural areas and agriculture is emphasized in the literature of the subject more and more often (Piwowar 2019b; Dzikuć et al. 2019; Bastan et al. 2018). This is particularly important in Poland due to the scale of agricultural activity (the area of agricultural land, livestock population, consumption of mineral fertilizers, etc.).

Rural areas are administratively 93.4% of Poland’s area (Górka 2018). Over the last 15–20 years, many structural changes took place in the agriculture in Poland, like in other countries of Central and Eastern Europe (Bański 2011; Grešlová et al. 2019). The changes concerned, inter alia, the number of agricultural holdings, land area, crops structure, and livestock population. In 2002, there were 2.9 million agricultural holdings in Poland, while in 2018, it was 1.4 million. The total area of the agricultural land in 2002 was 16.9 million ha, while in 2018, 14.7 million ha. In the structure of crops, cereals clearly predominate (approx. 70%). In the period of 2002–2018, the production of oilseed rape distinctly increased. A downward trend in this respect concerns root crops (mainly potatoes). Due to the concentration, very intensive methods are often used. These methods have a negative impact on the environment, including soil condition. Poland’s accession to the European Union (in 2004) and inclusion in the Common Agricultural Policy mechanisms not only caused changes in the agrarian structure but also increased the degree of equipping the agricultural holdings with means of production (fixed and current assets) (Smędzik-Ambroży et al. 2019). An unfavorable phenomenon from the point of view of environmental protection is an increase in the consumption of agrochemicals, especially mineral fertilizers. In 2018, the average consumption of mineral fertilizers in Poland was 141.6 kg NPK/ha agricultural land, compared to 102.4 kg NPK/ha in 2004. The literature of the subject more and more often emphasizes the negative impact of modern agriculture in Poland on the environment. This concerns, first of all, a reduction in the biodiversity, as well as intensive fertilization and the use of herbicides (Luque and Kostecka 2018; Piwowar 2019b; Wojciechowska et al. 2019). In turn, the number of organic farms in Poland is increasing, but they still represent a small share in the agrarian structure of the Polish agriculture (Moudrý et al. 2018; Biernat-Jarka and Trebska 2018).

The structural changes in the Polish agriculture and the challenges in the scope of low-carbon development prompted to undertake research aimed at learning the opinions and attitudes of farmers regarding actions and challenges in the field of environmental protection. The main purpose of this study is to assess the state of the natural environment in rural areas and to identify challenges associated with environmental protection in rural areas in a local context.

## Methodology and sources of information

This study was prepared in connection with a research project. The main sources of information used in it were results of questionnaire surveys. The spatial extent of the studies covered the whole Poland, while questionnaire surveys were conducted in six provinces in total (in each macroregion of Poland) on a sample of 1101 agricultural holdings.

The spatial extent of the project analysis conducted in the framework covered the entire country, while empirical research among farmers was conducted in six randomly selected provinces. Selection was based on localization research approach, based on territorial units. The study was conducted in six randomly selected provinces, one province from each of the macroregions in Poland—statistical units of the first level (NUTS 1). Random selection was also applied to districts within selected provinces. In each province, 3 districts had been drawn, in which the surveys were carried out.

Empirical studies among agricultural producers were carried out in the period from November 2017 to March 2018, using the questionnaire method and the sample selected. The sample was representative; i.e., it satisfied two conditions: each unit of the population had an equal chance to get into the sample, and the sample was sufficiently large. The exact size of the survey sample was estimated using the following formula (Sobczyk 1995; Suresh and Chandrashekara 2012):

$$ n\ge \frac{1}{4}{\left(\frac{u_{\frac{\alpha }{2}}}{d}\right)}^2 $$

where *n* is the minimum representative size of the sample, *u* is the critical value of the normal distribution, *d* is the maximum estimation error, and ${u}_{\frac{\alpha }{2}}$ is the normal deviate for two-tailed alternative hypothesis at a level of significance.

After substituting the formula, the number of observations should not be less than 1036. It has been assumed that the structure index will be estimated at the confidence level of 0.99 (*α* = 0.01). Statistical error was 4% (*d* = 0.04).

Field studies were carried out in the following provinces (the districts, in which the surveys were conducted, are given in parentheses):

- Lubelskie Province (Bialski, Lubartowski, and Zamojski districts);
- Małopolskie Province (Gorlicki, Proszowicki, and Tarnowski districts);
- Mazowieckie Province (Łosicki, Makowski, and Żuromiński districts);
- Opolskie Province (Kluczborski, Oleski, and Opolski districts);
- Pomorskie Province (Gdański, Kartuski, and Sztumski districts);
- Wielkopolskie Province (Gnieźnieński, Koniński, and Międzychodzki districts).

**Fig. 1:**
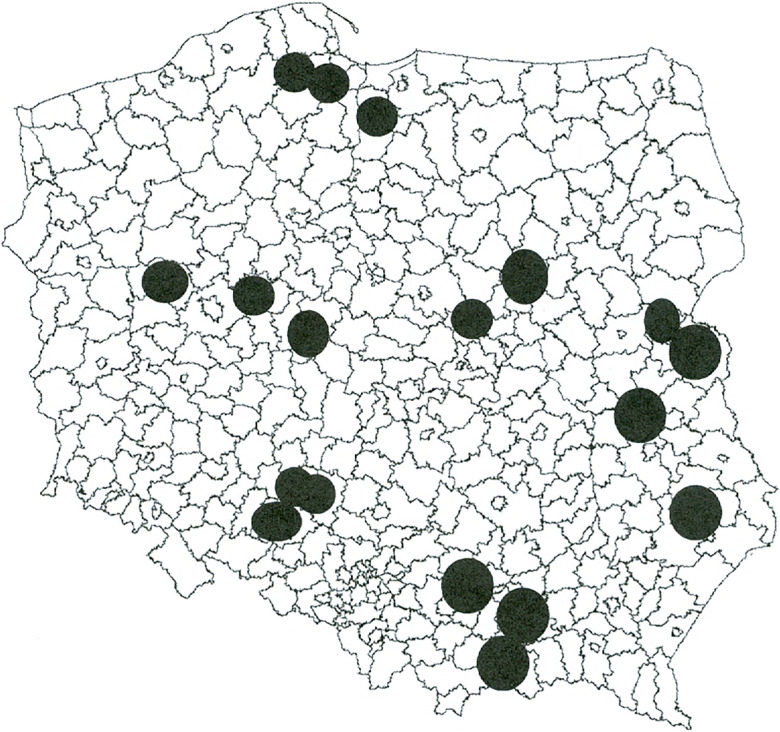
Spatial extent of the questionnaire surveys

The area of the research is marked in black in Fig. 1.

This paper presents selected results of the questionnaire survey concerning the farmers’ awareness of environmental protection in rural areas. The accuracy and reliability of the measurement with the use of the questionnaire was confirmed based on the method proposed by Cronbach (1951). The value of the coefficient obtained in relation to the questions analyzed in this study (Cronbach’s *α* = 0.923522830) is satisfactory, and the accuracy of questionnaire prediction can be considered very high.

The areas, in which the surveys were carried out, are characterized by a production potential that is relatively large on the scale of Poland and enables diversified agricultural production. Random selection of the test sample allowed conducting the surveys among agricultural producers dealing with both plant production and animal production. The total area of the agricultural holdings surveyed was 31,819.75 ha of agricultural lands, while the average area of an agricultural holding surveyed was 28.90 ha of agricultural lands. Arable lands predominated in the structure of agricultural lands of the agricultural holdings surveyed. A relatively high percentage of meadows and permanent pastures was recorded in Gorlicki, Żuromiński, Bialski, and Lubartowski districts. Basic data concerning the agricultural holdings surveyed and their owners are presented in Tables 1 and 2.

**Table 1:** General characteristics of the test sample (respondents)

Specification	Population (pcs)	Share in the test sample (%)
Age of respondents (years)		
18–29	128	11.6
30–39	250	22.7
40–49	328	29.8
50–59	281	25.5
> 60	102	9.3
No data	12	1.1
Total	1101	100
Gender of respondents		
Women	197	17.9
Men	901	81.8
No data	3	0.3
Total	1101	100
Education level of respondents		
Primary	44	4.0
Graduate vocational school	389	35.3
Secondary	518	47.0
Higher	142	12.9
No data	8	0.7
Total	1101	100
Number of years worked in agricultural holding		
1–5	90	8.2
6–10	146	13.3
11–15	119	10.8
16–20	172	15.6
21–25	138	12.5
26–30	152	13.8
> 31	282	25.6
No data	2	0.2
Total	1101	100

Source: own study based on questionnaire surveys (*N* = 1101)

**Table 2:** General characteristics of the test sample (selected features of agricultural holdings)

Specification	Population (pcs)	Share in the test sample (%)
Area of agricultural lands (ha)		
< 5	88	8.0
5–9.99	195	17.7
10–14.99	191	17.3
15–19.99	136	12.4
20–29.99	164	14.9
30–49.99	170	15.4
50–99.99	115	10.4
> 100	41	3.7
No data	1	0.1
Total	1101	100
Economic size of agricultural holding (SO), in euros		
< 10,000	316	28.7
10,100–13,000	156	14.2
13,100–20,000	188	17.1
20,100–50,000	232	21.1
50,100–100,000	99	9.0
100,100–200,000	40	3.6
> 200,000	4	0.4
No data	66	6.0
Total	1101	100

The sample size in selected provinces and districts is presented in the text of this paper. Source: own study based on questionnaire surveys (*N* = 1101)

The average age of respondents was 44 years, while the largest share in the age structure had the group of respondents aged 40–49 (almost 30% of the respondents). The participants in the surveys were mostly men (81.8 of the respondents who indicated their gender), while the majority of all the respondents had secondary education (47% of the respondents). Most respondents were characterized by a relatively considerable experience in agricultural activity. Over half of the respondents declared over 20-year work experience.

Agricultural holdings with an area of agricultural lands of 5–9.99 ha were the predominant group in the surveys (i.e., 17.7% of all agricultural holdings surveyed). Nevertheless, larger agricultural holdings were also represented by a significant number of respondents (in total, the agricultural holdings with over 30 ha of agricultural lands accounted for 44.5% of the entire test sample). In turn, the economic size of the surveyed agricultural holdings most frequently was below EUR 10,000 (28.7% of the agricultural holdings).

## Results of the research and discussion

One of the research questions was “What, in your opinion, are the most important factors affecting the condition of the natural environment in rural areas? Please indicate three most important factors.” The results of this part of the questionnaire survey in the spatial arrangement of the research are presented in Table 3.

**Table 3:** Declarations of respondents regarding the importance of selected factors affecting the condition of the natural environment in rural areas in the spatial arrangement of the research

Specification	Sample size (pcs)	1*	2*	3*	4*	5*	6*	7*
		%						
Lubelskie^a^	183	84.7	61.2	57.9	51.4	29.5	3.3	1.1
Bialski^b^	60	76.7	66.7	58.3	58.3	36.7	1.7	0.0
Lubartowski^b^	62	88.7	69.4	61.3	41.9	25.8	1.6	0.0
Zamojski^b^	61	88.5	47.5	54.1	54.1	26.2	6.6	3.3
Małopolskie^a^	188	78.2	70.7	54.8	49.5	30.3	4.8	0.0
Gorlicki^b^	68	86.8	70.6	52.9	54.4	30.9	1.5	0.0
Proszowicki^b^	60	70.0	71.7	51.7	43.3	31.7	6.7	0.0
Tarnowski^b^	60	76.7	70.0	60.0	50.0	28.3	6.7	0.0
Mazowieckie^a^	180	76.1	61.1	52.2	48.3	32.8	7.8	0.6
Łosicki^b^	60	66.7	66.7	31.7	63.3	23.3	3.3	1.7
Makowski^b^	60	83.3	68.3	81.7	28.3	31.7	5.0	0.0
Żuromiński^b^	60	78.3	48.3	43.3	53.3	43.3	15.0	0.0
Opolskie^a^	190	81.6	67.4	56.3	49.5	30.5	7.4	0.5
Kluczborski^b^	61	91.8	62.3	67.2	32.8	29.5	6.6	1.6
Oleski^b^	69	59.4	84.1	62.3	36.2	37.7	13.0	0.0
Opolski^b^	60	96.7	53.3	38.3	81.7	23.3	1.7	0.0
Pomorskie^a^	180	68.9	71.1	56.7	41.1	24.4	5.0	1.1
Gdański^b^	60	75.0	61.7	63.3	38.3	25.0	1.7	3.3
Kartuski^b^	60	50.0	75.0	58.3	35.0	16.7	13.3	0.0
Sztumski^b^	60	81.7	76.7	48.3	50.0	31.7	0.0	0.0
Wielkopolskie^a^	180	68.9	66.1	51.	43.3	22.8	13.9	2.2
Gnieźnieński^b^	60	78.3	68.3	51.7	46.7	16.7	16.7	3.3
Koniński^b^	60	70.0	66.7	58.3	40.0	25.0	13.3	1.7
Międzychodzki^b^	60	58.3	63.3	45.0	43.3	26.7	11.7	1.7
Total	1101	76.5	66.3	55.0	47.2	28.4	7.0	0.9

Source: own study based on questionnaire surveys (*N* = 1101)

^a^Voivodeships

^b^Districts

*For 1, responsibility of farmers and other residents of rural areas; 2, subsidies for replacement of boiler heaters, thermomodernization, etc.; 3, financial support for key agri-environmental practices (e.g., from the rural development program); 4, activity of local authorities in the scope of environmental protection (development of strategies and plans in this respect); 5, activity of authorities at the central level (including that in the scope of ecological education); 6, hard to tell; and 7, other

According to the analyses, the factor “responsibility of farmers and other residents of rural areas” obtained most “yes” answers. For 76.5% of the respondents, this is an important factor that determines the condition of the natural environment in rural areas in Poland to the greatest extent. Important factors included also “subsidies for replacement of boiler heaters, thermomodernization, etc.” (indications of 66.3% of the respondents); “financial support for key agri-environmental practices (e.g. from the Rural Development Programme)” (indication of 55% of the respondents); and “activity of local authorities in the scope of environmental protection (development of strategies and plans in this respect)” (indication of 47.2% of the respondents). The respondents indicated that the factor “activity of authorities at the central level (including that in the scope of ecological education)” is much less important. Seven percent of the respondents did not have any opinion on this topic, while less than 1% of the respondents indicated a different factor.

Taking into account the spatial extent of the analyses, significant differences in respondents’ indications were found. For example, the respondents from Opolski District (96.7% of the respondents) declared that the responsibility of farmers and other residents of rural areas with respect to the examined issues was very important, while in Kartuski District, this factor was indicated by only half of the respondents. Large disparities were also observed in the analysis of other factors:

- The factor “subsidies for replacement of boiler heaters, thermomodernization, etc.” was important for 84.1% of the respondents from Oleski District (on average, this factor was indicated by 66.3% of all respondents participating in the survey);
- The factor “financial support for key agri-environmental practices (e.g. from the Rural Development Programme)” was important for 81.7% of the respondents from Makowski District (on average, this factor was indicated by 66.3% of all respondents participating in the survey);
- The factor “activity of local authorities in the scope of environmental protection (development of strategies and plans in this respect)” was important for 81.7% of the respondents from Opole District (on average, this factor was indicated by 66.3% of all respondents participating in the survey).

In addition, many respondents, especially from Wielkopolskie Province, gave the answer “hard to tell” to the question formulated in such a way. On average, 13.9% of the respondents from this province answered in such a way, including:

- 16.7% of the respondents from Gnieźnieński District;
- 13.3% of the respondents from Koniński District;
- 11.7% of the respondents from Międzychodzki District.

A high percentage of such responses was also recorded in Żuromiński (15%), Oleski (13.0%), and Kartuski (13.3%) districts.

Tables 4 and 5 present results of this part of the research, taking into account the features of the respondents and agricultural holdings.

**Table 4:** Declarations of respondents regarding the importance of selected factors affecting the condition of the natural environment in rural areas, taking into account demographic and social features of the respondents

Specification	Sample size (pcs)	1*	2*	3*	4*	5*	6*	7*
		%						
Age of respondents (years)								
18–29	128	78.1	64.8	65.6	39.8	27.3	5.5	1.6
30–39	250	78.0	66.4	60.8	48.8	30.0	5.6	0.4
40–49	328	76.2	63.4	53.4	48.2	26.2	7.9	1.2
50–59	281	77.2	71.2	49.1	50.2	30.2	6.0	0.4
> 60	102	71.6	62.7	54.9	43.1	29.4	10.8	2.0
Gender of respondents								
Women	197	77.2	68.5	58.9	48.7	31.5	6.1	0.5
Men	901	76.4	65.7	53.9	46.9	27.9	7.2	1.0
Education level of respondents								
Primary	44	56.8	68.2	45.5	40.9	25.0	15.9	2.3
Graduate vocational school	389	72.8	69.7	49.9	47.0	28.0	9.3	0.5
Secondary	518	79.9	66.4	59.1	47.7	26.8	4.8	1.0
Higher	142	79.6	59.9	57.0	48.6	35.9	6.3	1.4
Number of years worked in agricultural holding								
1–5	90	72.2	64.4	60.0	47.8	34.4	7.8	1.1
6–10	146	80.1	66.4	62.3	46.6	28.1	6.2	0.7
11–15	119	79.8	63.0	60.5	48.7	31.1	5.0	0.8
16–20	172	79.1	70.3	66.3	40.7	19.2	5.8	0.6
21–25	138	78.3	65.9	52.9	45.7	29.7	9.4	0.7
26–30	152	71.1	65.1	48.7	49.3	28.9	8.6	1.3
> 31	282	75.5	60.6	41.8	47.5	29.4	6.7	1.1

Source: own study based on questionnaire surveys (*N* = 1101)

*For 1, responsibility of farmers and other residents of rural areas; 2, subsidies for replacement of boiler heaters, thermomodernization, etc.; 3, financial support for key agri-environmental practices (e.g., from the rural development program); 4, activity of local authorities in the scope of environmental protection (development of strategies and plans in this respect); 5, activity of authorities at the central level (including that in the scope of ecological education); 6, hard to tell; and 7, other

**Table 5:** Declarations of respondents regarding the importance of selected factors affecting the condition of the natural environment in rural areas, taking into account features of the agricultural holdings surveyed

Specification	Sample size (pcs)	1*	2*	3*	4*	5*	6*	7*
		%						
Area of agricultural lands (ha)								
< 5	88	72.7	64.8	54.5	44.3	34.1	10.2	0.0
5–9.99	195	71.3	71.8	57.4	50.8	26.7	4.6	1.0
10–14.99	191	75.4	67.0	56.0	42.4	28.8	6.3	0.0
15–19.99	136	72.8	69.1	57.4	49.3	23.5	7.4	0.0
20–29.99	164	76.2	64.0	52.4	46.3	31.7	9.8	3.0
30–49.99	170	80.6	64.7	52.4	48.2	28.8	7.1	0.6
50–99.99	115	85.2	61.7	53.0	49.6	30.4	3.5	1.7
> 100	41	85.4	61.0	58.5	46.3	19.5	12.2	0.0
Economic size of agricultural holding (SO), in euros								
< 10,000	316	74.4	67.7	57.3	44.3	28.8	7.0	0.9
10,100–13,000	156	75.6	64.7	58.3	46.8	30.8	5.1	0.0
13,100–20,000	188	72.3	69.1	56.4	42.6	26.1	11.2	2.1
20,100–50,000	232	80.6	68.5	51.3	48.7	31.5	6.5	1.3
50,100–100,000	99	78.8	64.6	57.6	56.6	31.3	4.0	0.0
100,100–200,000	40	92.5	57.5	52.5	50.0	17.5	5.0	0.0
> 200,000	4	75.0	75.0	25.0	50.0	50.0	25.0	0.0

Source: own study based on questionnaire surveys (*N* = 1101)

*For 1, responsibility of farmers and other residents of rural areas; 2, subsidies for replacement of boiler heaters, thermomodernization, etc.; 3, financial support for key agri-environmental practices (e.g., from the rural development program); 4, activity of local authorities in the scope of environmental protection (development of strategies and plans in this respect); 5, activity of authorities at the central level (including that in the scope of ecological education); 6, hard to tell; and 7, other

The answers of the respondents did not differ based on their gender. As it appears from the analyses, there were recorded relatively similar responses regarding the importance of these factors, taking into account the age of the respondents. The only exception is the group of people over 60 years of age (102 persons) who attached relatively less attention to factors 1 and 3. A significant percentage of the respondents in this age group gave the answer “hard to tell.” Considerable differences in indications of the respondents were observed, taking into their experience and level of education. People with primary education more often gave the answers “hard to tell” and attached less attention to factor 1. In turn, people with higher education definitely more frequently declared that “activity of authorities at the central level (including that in the scope of ecological education)” is necessary. The level of education clearly differentiated responses regarding the importance of factor 1. Considering the experience in work in agriculture, the people with work experience up to 20 years declared that “subsidies for replacement of boiler heaters, thermomodernization, etc.” was an important factor more frequently than respondents with more work experience.

Features of agricultural holdings (surface area and economic size) also differentiated responses regarding the factors affecting the condition of the natural environment in rural areas. It was particularly distinctive that more attention was paid to factor 1 and less to factor 2 in the group of agricultural holdings with over 30 ha of agricultural lands (as compared with smaller agricultural holdings). In addition, owners of agricultural holdings with an economic size of over EUR 50,000 declared a relatively greater significance of factor 4.

According to the analyses, the desired condition of the natural environment in rural areas in Poland can be achieved through its appropriate use, protection, and shaping. Economic practices and full acceptance of local communities with regard to balancing the social, economic, and natural aspects are of fundamental importance in the evolution of the Polish agriculture towards carbon efficiency and sustainability. It is necessary not so much to accelerate changes in farmers’ awareness but to implement appropriate practices in the scope of nature conservation and use of biological resources in a sustainable manner (precision farming, etc.). A relatively high level of awareness among Polish farmers in the scope of the natural environment is also presented in other publications (Kaczocha and Sikora 2016; Sulewski and Gołaś 2019).

The literature of the subject emphasizes that the agriculture in Poland has a vital role to play in the fight against climate change. The scale of the development of the agriculture and forestry in Poland makes it possible to further increase the capacity to absorb carbon dioxide. However, it is necessary to invest in modernization and innovation as well as in new research and development of techniques aimed at adaptation to climate change. Opportunities can be seen, inter alia, in the development of renewable energy sources and production of biomass for non-food purposes (Piwowar and Dzikuć 2019; Zyadin et al. 2019; Loizou et al. 2019; Czekała et al. 2020). There is a need for appropriate local programs to support these solutions as well as instruments that encourage the provision of services for the benefit of the environment in line with public demand. The problem is the coordination of implementation in spatial development plans at the national, provincial, and communal levels, which hinders or even prevents the integration and development of rural areas (Woch and Jędrek 2016). Reducing the deficiency in the diversity of species and landscape in rural areas is another important task from the point of view of natural environment and its protection. This also requires ongoing monitoring and responding to new challenges at the local and regional levels. Thermomodernization is also an important and topical issue in rural areas in Poland. This is a very important element reducing the phenomenon of energy poverty in rural areas. During the studies, large heat losses caused by a lack of thermal insulation of external walls and basement walls, roofs of buildings, poor technical condition of the windows, etc., were found. Farmers notice this problem in relation to residential and livestock buildings (Piwowar 2019a). Agriculture in Poland is also a significant source of greenhouse gas emissions (Wiśniewski and Kistowski 2019).

## Summary

Relations between the agricultural socioeconomic system and the natural environment in Poland are the subject of considerations in many scientific fields and disciplines. Based on the analyses, it can be concluded that agricultural producers in Poland are aware of environmental threats posed by irrational agricultural economy. They are able to evaluate factors affecting the condition of the natural environment in rural areas, taking into account nationwide, regional, and local problems. According to the analyses, agricultural producers indicated various factors depending on the location of their agricultural holding. Nevertheless, the responsibility of farmers and other residents of rural areas is a basic condition for broadly understood environmental safety in rural areas in Poland. In addition, systems for funding the replacement of heating boilers, thermomodernization of buildings, etc., are also important. Financial support for key agri-environmental practices is of considerable importance. The differentiation of responses in the scope of these factors was determined by the age and level of education of a given respondent. Features of agricultural holdings, i.e., the surface area and economic size, were also important in terms of differentiation of respondents’ indications.

The studies carried out allowed highlighting the role of local institutions and activities, as well as the specific social and environmental features of the selected regions in the context of protection of the natural heritage in rural areas of Poland. The investigated subject matter is related to multi-layered problems. However, this is becoming increasingly important, considering climate change and the need to minimize the negative impacts. For the sustainable development of agricultural ecosystems, it is particularly important to increase the social responsibility of farmers. The relationship between a farmer and the environment is the most important subject of considerations in this respect.
